# Comparisons of neurodegeneration over time between healthy ageing and Alzheimer's disease cohorts via Bayesian inference

**DOI:** 10.1136/bmjopen-2016-012174

**Published:** 2017-02-07

**Authors:** Marcela I Cespedes, Jurgen Fripp, James M McGree, Christopher C Drovandi, Kerrie Mengersen, James D Doecke

**Affiliations:** 1 School of Mathematical Sciences, Queensland University of Technology, Brisbane, Queensland, Australia; 2 CSIRO Digital Productivity and Services, Australia E-Health Research Centre, Herston, Queensland, Australia

**Keywords:** Bayesian inference, mixed effects models, neurodegeneration, Alzheimer's disease, longitudinal neuroimaging study

## Abstract

**Objectives:**

In recent years, large-scale longitudinal neuroimaging studies have improved our understanding of healthy ageing and pathologies including Alzheimer's disease (AD). A particular focus of these studies is group differences and identification of participants at risk of deteriorating to a worse diagnosis. For this, statistical analysis using linear mixed-effects (LME) models are used to account for correlated observations from individuals measured over time. A Bayesian framework for LME models in AD is introduced in this paper to provide additional insight often not found in current LME volumetric analyses.

**Setting and participants:**

Longitudinal neuroimaging case study of ageing was analysed in this research on 260 participants diagnosed as either healthy controls (HC), mild cognitive impaired (MCI) or AD. Bayesian LME models for the ventricle and hippocampus regions were used to: (1) estimate how the volumes of these regions change over time by diagnosis, (2) identify high-risk non-AD individuals with AD like degeneration and (3) determine probabilistic trajectories of diagnosis groups over age.

**Results:**

We observed (1) large differences in the average rate of change of volume for the ventricle and hippocampus regions between diagnosis groups, (2) high-risk individuals who had progressed from HC to MCI and displayed similar rates of deterioration as AD counterparts, and (3) critical time points which indicate where deterioration of regions begins to diverge between the diagnosis groups.

**Conclusions:**

To the best of our knowledge, this is the first application of Bayesian LME models to neuroimaging data which provides inference on a population and individual level in the AD field. The application of a Bayesian LME framework allows for additional information to be extracted from longitudinal studies. This provides health professionals with valuable information of neurodegeneration stages, and a potential to provide a better understanding of disease pathology.

**Strengths and limitations of this study**

- The models presented in this research address realistic challenges in a longitudinal study setting such as: large patient drop-out (unbalanced design), large and small diagnosis groups and noisy MRI observations.
- This is the first study of its kind to incorporate data external to this analysis, in terms of prevalence rates, in conjunction with the statistical models to infer disease trajectories for brain regions over age.
- This research does not accommodate participants with other neurological disorders and assumes that participants are in one of three groups: healthy control, mild cognitive impaired and Alzheimer's disease.
- Additional covariates which are known to affect neurodegeneration were not included in this analysis, such as gender and genetic status.

## Introduction

Alzheimer's disease (AD) is the most common form of dementia worldwide.^1^ Advances of neuroimaging techniques have been useful for early diagnosis of neurodegenerative disorders^2^ ^3^ and, coupled with mathematical and statistical models, provide insight to better understand healthy ageing and disease pathology degeneration.^4–6^ The use of linear mixed-effects (LME) models has been advocated by Bernal-Rusiel *et al*^7^ and, recently, by Ziegler *et al*^8^ to characterise longitudinal degeneration from neuroimaging data. Bayesian LME (BLME) models are applied in this research to provide insight into the diagnosis of AD over time. In this research, we address three main areas: population diagnosis comparisons based on estimated volumetric rate of change over age, ranking of participants by order of linear volumetric rate of change and region-specific probability trajectories across age of diagnosis groups, conditional on prevalence rates.

Recent state-of-the-art analysis on clinical diagnosis classification groups emphasises the need to better understand disease pathology in asymptomatic and early stages of individuals with AD.^9–13^ A strong focus of longitudinal neuroimaging studies is to monitor morphological changes among healthy control (HC), mild cognitive impaired (MCI) and AD groups as they progress throughout the disease continuum.^7^ ^14^

Previous LME models of volumetric degeneration reported on comparisons assessing ranking of diagnosis levels.^7^ ^15^ However, in these studies, the magnitude of the differences of disease progression as well as their estimated variances is often excluded;^7^ ^14–17^ thus, a richer insight into the differences of diagnosis levels is lacking. The BLME approach uses simulation techniques to draw from the posterior distribution, which is a combination of prior information and information from the data (through the likelihood function), to provide diagnosis group estimates and comparisons. These simulations quantify uncertainty and provide posterior probabilities that can be compared directly, without referring to significance levels or multiple statistical tests.

The development of methods which account for large intervariability and intravariability of biomarkers presents a challenge in longitudinal neuroimaging studies.^18–20^ Furthermore, the observations of diagnosis group tends to become unbalanced over time, which makes it difficult to deduce information of the complex AD pathway. However, insight into neurodegeneration of high-risk participants, namely MCI, is crucial for early detection methods and improving diagnostic accuracy of AD.^5^ ^21^ Several authors such as Harville and Carriquiry,^22^ Gelman and Hill^23^ and Li *et al*^24^ state that BLME models have the capability to seamlessly handle unbalanced data and small-sample design analysis. This motivates our choice of statistical framework, as we aim to use as much information as possible from the study analysed and retain participants with a single observation.

Individuals in order of neurodegeneration severity allow for comparisons of progression of all individuals over the study, while quantifying the uncertainty and estimating variability of individualised conversion rates. The application of BLME models allows for estimation of class membership probabilities and estimation of deterioration rates of each participant via the analysis of random effects. This type of analysis is often overlooked in longitudinal studies of ageing.^25^

Since the field of neuroimaging in AD has been rapidly expanding in the past 20 years,^18^ ^26–28^ it is of interest to incorporate as much relevant information as possible, as independent longitudinal neuroimaging studies often build on and support each other.^5^ ^8^ ^29^ This can be achieved using the Bayesian approach, as it combines external information with experimental data at hand, while accounting for various sources of uncertainty. This background information can often be incorporated in the form of the prior, but it can also be applied after estimation of the model to provide additional inference from our model outcomes. In the current project, we demonstrate this concept by combining model information with prior knowledge obtained from prevalence studies to formulate probabilistic diagnosis group trajectories over age.

Jack *et al*^30^ highlight the importance of population frequency or probabilistic trajectories of neurodegeneration groups over a wide age span. Their study quantified frequencies of expected neurodegeneration cases dependent on ages 50–89. Particular focus was placed on asymptomatic individuals (preclinical AD) who were at risk of developing AD and ages of increased frequency of convergence to AD as they reach their later years. While our methods can also be used for similar purposes and place emphasis on a particular neurodegeneration group, the goal for our final analysis is to identify critical time points where all diagnosis levels begin to diverge. This can aid in discovering groups or patterns in neurodegeneration consistent with healthy ageing or the AD pathway. Alternatively, a similar analysis can also be used to compare diagnosis trajectories of different longitudinal neuroimaging population studies, such as the Alzheimer's Disease Neuroimaging Initiative (ADNI).

This paper is outlined as follows. The AIBL longitudinal study of ageing section describes the case study. The How do HC, MCI and AD participants degenerate over time? sections show an application of the BLME models to address multiple comparisons of various sizes from baseline diagnosis, including large (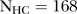 people) and small groups (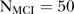
and 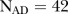 people at baseline). The How to identify individuals with high levels of neurodegeneration? sections rank individuals by order of neurodegeneration severity, thereby comparing the progression of all individuals over the study time. Approximately 10% of individuals convert from a baseline case to a worse diagnosis throughout the length of the study. This analysis allows for the identification of those participants who are most at risk of developing AD like rates of deterioration for the hippocampus and ventricle regions of the brain. The third and final area addressed in this research is presented in the How do diagnosis trajectories vary over age? sections, which estimates probabilistic diagnosis group trajectories across age, derived from neuroimaging information. This requires the synthesis of information from the study cohort and the AD literature.

## AIBL longitudinal study of ageing

The neuroimaging data analysed in this paper were obtained from the Australian Imaging Biomarker and Lifestyle Study of Ageing (AIBL). This is an ongoing study which aims to discover which biomarkers such as cognitive assessment results, neuroimaging, lifestyle and demographic factors potentially influence subsequent development of AD. The sample comprises 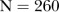 people, who have at most four repeated observations ∼18 months apart. These data are highly unbalanced, since patient drop-out occurs at every time point throughout the study, with ∼69% of participants in the final follow-up.

Key regions of the brain which are strongly associated with neurodegeneration in relation to AD and healthy ageing include the lateral ventricles^31^ ^32^ and hippocampus volumes.^3^ ^15^ ^33–35^ Atrophy due to disease pathology spreads throughout particular regions such as the hippocampus, which leads to a general decrease in volume over time. The decrease in brain matter results in an increase in cerebrospinal fluid (CSF) which bathes and cushions the brain and spinal cord. The lateral ventricles are filled with CSF; hence, an increase in overall brain atrophy results in an increase in ventricle volume. Models presented here were considered separately for the lateral ventricles and the sum of the left and right hippocampal (hippocampus) volumes derived from MRI. See Rowe *et al*^36^ for details on image acquisition and processing. While we cannot deduce entire brain neurodegeneration inferences from the analysis of two regions, in this research we discuss in detail the application of two well-known AD-related regions and note that the BLME models presented here can be easily applied to any other region of interest.

Brain region volumes were normalised by the intracranial volume (ICV); hence, all volumes are in the (0, 1) interval. This accounts for the variability of different cranial sizes, while preserving the trend in volume.^37^ ^38^ Owing to the wide range in values and in order to eliminate numerical problems in the estimation of these models, age was standardised 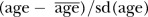, where 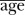 and 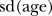 are the empirical mean and standard deviations of the study group ages. Likewise, the hippocampus ICV response was scaled up by a factor of 100, in order to avoid variance estimates close to 0 which can be difficult to estimate. All participants in this study were categorised as: HC, MCI and those with a probable diagnosis of AD at each time point based on neuropsychological diagnosis. The aim of the BLME models was to capture the linear decrease in regional brain volume across ages for people within three diagnosis groups.

## Methods

LME models are a standard approach to modelling repeated observations from several individuals.^39^ Standard LME models require the following assumptions to be met: a linear relationship exists between the response and the explanatory variables; the terms at every level are Gaussian, although for non-normal models we may extend this assumption to the exponential family and apply generalised linear mixed models;^40^ the variances across all levels are homoscedastic, and repeated observations for an individual can be correlated, but observations between people are assumed to be independent. The general LME model is of the following form:1
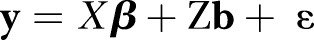


where 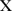 and 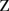 denote the design matrices, and vectors 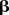 and 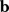 are the fixed and random effects, respectively, for 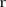 fixed, 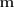 random effects and a total sample size of 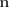 observations. The residual vector 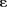 is assumed to be normally distributed with 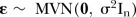, where 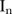 is the 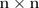 identity matrix. While our response values are constrained to the (0, 1) range, the assumptions of the model were assessed via a histogram of the residuals, scatter and quantile–quantile plots and were found to not deviate from our model assumptions (refer to the online supplementary material). The parameters in this analysis are in the volume ICV/standard age unit and careful back transformation is required to convert to an alternative unit, such as mm^3^/year. The random-effects vector 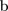 is assumed to be multivariate normally distributed, 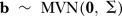, where the variance–covariance matrix of the random effects is denoted by Σ.

### Statistical analysis

In a Bayesian framework, the likelihood corresponding to the model in equation (1) is 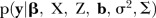, which is conditional on the random effects and on the model parameters. The resultant joint posterior distribution for the model parameters and random effects given in the data is 2
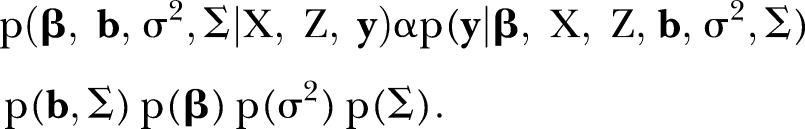


In the absence of external information, weakly informative priors, 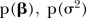 and 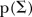, were used throughout (refer to equation (3) in the BLME in the context of the case study section for full specification of priors). Under the Bayesian paradigm, all the assumptions stated in the Methods section remain. Furthermore, as Gelman *et al*^41^ and Gelman and Hill^23^ state, additional complexity and generalisation of the LME model comes naturally under the Bayesian framework.

Estimation of the model parameters was achieved by sampling from the joint posterior distribution using Markov chain Monte Carlo (MCMC) techniques^42^ which samples from the marginal posterior distributions as a by-product. Note that the parameter estimates are obtained by integrating over the posterior distribution, rather than maximising the likelihood, as numerical methods to solve integrals in high dimensions are often difficult to compute.^42^ ^43^

### BLME in the context of the case study

Following equation (1), the normalised volume is denoted by 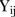 for the 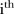 individual at the 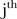 time point, where binary values 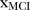 and 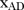 refer to the two levels of diagnosis, MCI and AD, respectively, with HC as the baseline. The BLME model for person 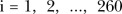 at time point 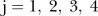 is given by3
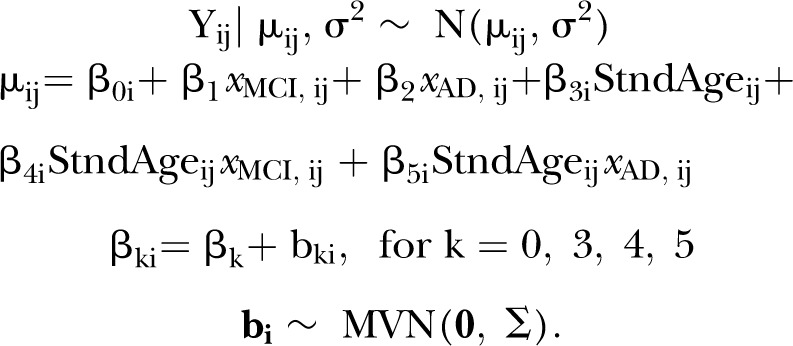


Random effects 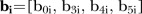 denote the 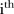 individuals deviation from population means 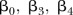 and 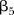. The model in equation (3) allows for correlation between random effects and this is reflected by the structure of the priors. The variance of the residual and the variance–covariance matrices are designated by semiconjugate priors 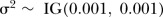 and 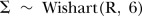, respectively, where 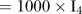. The fixed-effects vector 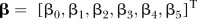 is assumed to be normal with 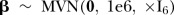. Non-linear trends in age were investigated in order to derive an appropriate model for our application (refer to the online supplementary material for further details). However, the linear predictor in equation (3) was found to approximately represent the data. Posterior predictive plots were used as a measure of goodness-of-fit. This involved simulating from the posterior distribution and forming 95% credible intervals of the posterior predictive responses, which were compared with the observed responses.

The R software was used to implement the Bayesian models.^44^ The rjags package^45^ implemented MCMC methods to estimate the parameters. Packages coda^45^ and ggplot2^46^ were used to analyse the MCMC chains and visualise the three sets of analyses presented here. All R source code for this manuscript and simulated data is available at github website https://github.com/MarcelaCespedes/Bayesian_inference_on_neuroimaging.

Two independent MCMC runs were performed using different starting values; each chain ran for 300K iterations of which 100K were discarded as burn-in and the remaining simulations were thinned at every 50th iteration. The retained 8000 simulations were taken as samples from the posterior distribution. Convergence diagnostics of the chains included observing the trace, density and autocorrelation plots as well as the Gelman and Rubin^47^ diagnostic. Desirable chain mixing and convergence was observed in all diagnostics. In addition to the residual and posterior checks, leave-one-out cross-validation (LOOCV) was performed to assess the model’s predictive capability of new data, and the mean squared error (MSE) was computed on both models. In a hierarchical setting, the size of the data and how balanced the structure is heavily affects the relative performance of the model.^48^ For this reason, we performed two approaches for LOOCV on the ventricle and hippocampus models. First, all the observations for an individual were omitted from the analysis (and, therefore, all of their data), and this was repeated for all individuals. Second, for those participants with more than one observation (199 participants in our data set), a single observation was randomly removed from the analysis (refer to the online supplementary material for full results). In practice, we wish to minimise the MSE, as it comprises the sum of the variance, bias squared and irreducible error. Both LOOCV approaches demonstrated low MSE values, which support our model choice (refer to the online supplementary material for full details).

For comparison, the research questions addressed here were attempted with the model in equation (3) fitted in the classical framework for both regions. The How do diagnosis trajectories vary over age? and How to identify individuals with high levels of neurodegeneration? sections discuss the results for each analysis.

### M: How do HC, MCI and AD participants degenerate over time?

Performing a Bayesian analysis provides a posterior distribution of the parameter which can be used here to estimate the rate of volumetric degeneration for each diagnosis level.^16^ In this analysis, we estimate a diagnosis group effect via the posterior mean of the relevant parameter, and investigate differences in these effects via credible intervals (about differences of these means). Other than mean diagnosis comparisons, further analysis in terms of mean differences of these groups is often not performed in LME volumetric neuroimaging models.^7^ ^49^ However, as highlighted in Apostolova *et al*^31^ and Holland *et al*,^15^ such insight allows for potential techniques to detect signs of AD like neurodegeneration on presymptomatic individuals.

As indicated in equation (3), the population rate of deterioration for each diagnosis consists of the addition of the baseline effect (HC) with the interaction terms for the other diagnosis groups (MCI or AD). Thus, the posterior marginal distributions of 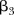 for the baseline, 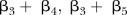, for MCI and AD diagnosis, respectively, were compared.

Furthermore, the order of deterioration of the diagnosis levels over both brain regions was assessed. Posterior probabilities were used to order parameter values, since this allows for direct probabilistic diagnosis group comparisons based on the MCMC output while quantifying uncertainty in the parameter estimates. Let M be the number of MCMC posterior draws; in our methods, M=8000 as described in the BLME in the context of the case study section.

The probability that the rate of change for MCI degeneration is smaller than an AD diagnosis for the ventricle region is estimated by 4
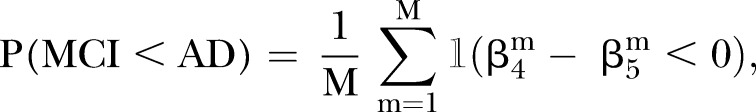
where the indicator function 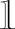 is equal to 1 if 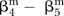 <0 and 0 otherwise. Probabilities for other comparisons of diagnosis levels for the ventricle and hippocampus regions are computed in a similar manner; see the R: How do HC, MCI and AD participants degenerate over time? section for full results.

### R: How to identify individuals with high levels of neurodegeneration?

It is expected that individuals who are healthy (HC) will have relatively minimal deterioration while those with MCI or AD will show increasing levels of deterioration. Hence, we would expect that the volumetric rate of change will reflect the neuropsychological clinical diagnosis. However, as noted by Woolrich *et al*,^50^ Bernal-Rusiel *et al*^7^ and Bernal-Rusiel *et al*,^51^ high intervariability and intravariability is often observed in longitudinal neuroimaging studies. For this reason, in this analysis we foresee the estimated volumetric rate of change for a few individuals not to group with participants of the same diagnosis and exercise caution when comparing estimated trajectories of individuals with a single observation.

Participants with outlier rates of deterioration or not within range of their diagnosis levels, as well as those who converted throughout the study, are of particular interest as they do not conform to the overall trend over time ordering. Thus, a question of interest might be: If an individual has a high neurodegeneration rate with respect to their corresponding diagnosis group, are they likely to degenerate along the AD pathway?

In our data, 1 individual progressed directly from HC to AD, 2 were observed to follow the full spectrum (HC to MCI to AD throughout all 4 follow-ups), 8 people progressed from HC to MCI and a further 16 individuals progressed from MCI to AD. These converters can be tracked to observe their severity with respect to the rest of the cohort. In this section, particular focus is on the converters who progressed from HC to MCI, and the comparison of their estimated rates of deterioration with AD participants, as they could be potential AD converters and estimate their probability of remaining in such a high rank.

Unlike our first analysis, which compared the estimated population effect across all diagnosis levels, the focus here is on an individual's rate of deterioration. The marginal posterior distributions of individual random-effects values in the HC 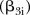, MCI 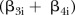 and AD 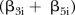 groups are inspected, to estimate the rate of deterioration for 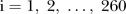 individuals on all four time points.

Furthermore, as discussed in the R: How to identify individuals with high levels of neurodegeneration? section and shown in the ordered box plots, the median rankings of participants and illustrate the large variation between individuals. Distribution of ranks on participants takes into account the high variation between individuals, by ranking participants at every iteration of the MCMC simulation of the random effects. This results in M=8000 simulations on every individual and allows us to derive probabilistic statements on individuals of interest remaining in a specified ranking range, for example, the top 15th quantile. This analysis was performed on both a subset of the data, using observations with the first three time points, as well as on the full data (four time points) to investigate the change of rank probabilities over time for particular individuals of interest. Such analysis extends the BLME models to allow for the identification of high-risk converters among the participants analysed. Full results are described in the R: How to identify individuals with high levels of neurodegeneration? section.

### M: How do diagnosis trajectories vary over age?

The ventricle and hippocampus models derived in equation (3) were used to compute probabilities 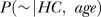, 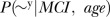 and 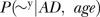, for a specified age with volume range denoted by 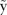. Given the information available on an individual at an early age and within the limits of our data age span, we seek to answer: At this early age, for a given volume range, what is the probability that this new individual will be diagnosed as HC, MCI or AD? Moreover, how does this change as the individual ages? These probabilities are estimated below.

At a given age for ventricle and hippocampus models stated in equation (3) with diagnosis levels 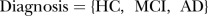, the following holds:5
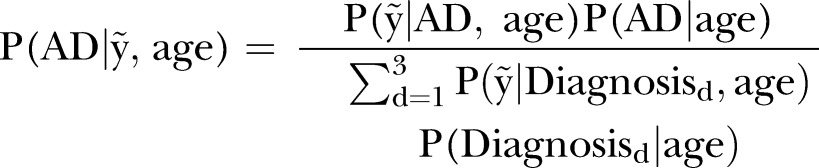


**Figure 1:**
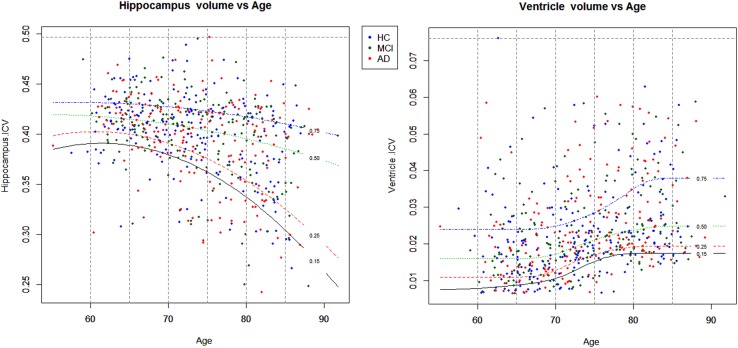
Centile ranges of volume across ages 60–85 years, for ventricle (left) and hippocampus (right). Recall region volumes are normalised by the ICV value as they represent a percentage of volume within the intracranial cavity. Ranges up to the 100th centile henceforth denote the empirical maximum volume for that region. Volume centiles; 75–100th from blue (0.75) to top dotted line, 50–75th from green (0.25) to blue (0.75) line, 25–50th from red (0.25) to green (0.50) line and 15–25th from black (0.15) to red (0.25) line. AD, Alzheimer's disease; HC, healthy control; ICV, intracranial volume; MCI, mild cognitive impaired.

The BLME model estimates 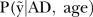, 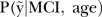 and 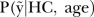. Since M is the number of MCMC posterior draws,6
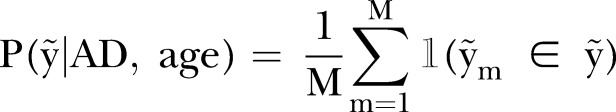


where the indicator function 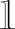 is equal to 1 if 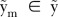 and 0 otherwise. This expression is the average number of predicted values 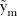 which fall within 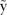. A similar expression was used for MCI and HC diagnosis levels. Probabilities 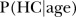, 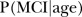 and 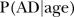 were obtained from Ward *et al*^52^ and Refshauge and Kalisch^53^ for ages 60, 65, 70, 75, 80 and 85. We acknowledged that these are very broad estimates which are generalised over genders, genetic status and many other factors which are known to affect prevalence rates. These prevalence rates also do not take into account participants who develop other forms of dementia, or any other neuropsychological disorders. Refer to the online supplementary material for the full table of probabilities used in this analysis. Similar computations were performed for the other diagnosis levels, MCI and HC, to evaluate related probabilities. Owing to the wide variability observed in the hippocampus and ventricle volumes among participants, the volume regions were divided into four different ranges, 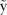, which vary over age groups. Quantile growth curves discussed in Cole and Green,^54^ and Koenker^55^ highlight the advantages of algorithms that can estimate non-crossing quantiles which are monotone increasing over age to reflect the heteroscedasticity often found in biological systems. In this paper, we used the algorithm discussed in Muggeo *et al*,^56^ as it addresses all of these issues and is available via R package quantregGrowth. The 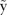 values of took on ranges; 75–100th, 50–75th, 25–50th and 15–25th centiles of observed response values, as shown in figure 1.

For completeness in our analysis, volume ranges such as the 5–25th centile were explored. However, there was very little difference in the probability trajectories among these volume ranges; hence, we maintained the 15–25th centile range. Furthermore, we wished to avoid low volume outliers, and place emphasis on the degenerating trends present in the majority of the data, for biologically meaningful inferences.

The results from applying equation (5) show probability trajectories of an individual being in one of the three diagnosis levels, across ages 60–85 within the four quantile ranges. The goal for this analysis is to identify critical time points where diagnosis levels begin to diverge which can aid in discovering groups or patterns in neurodegeneration consistent with healthy ageing or the AD pathway. Furthermore, the influence of covariates gender and apolipoprotein-E (APOE) was explored by repeating this analysis on subgroups of male, female, APOE positive and negative.

A similar analysis cannot be performed with a classical LME model, as the method of maximation of the likelihood does not allow for the straightforward computation of probabilities 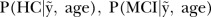 and 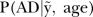. Another drawback of the classical approach is that it does not lend itself to the incorporation of relevant additional external data, to further extend statistical inference.

## Results

### R: How do HC, MCI and AD participants degenerate over time?

**Figure 2:**
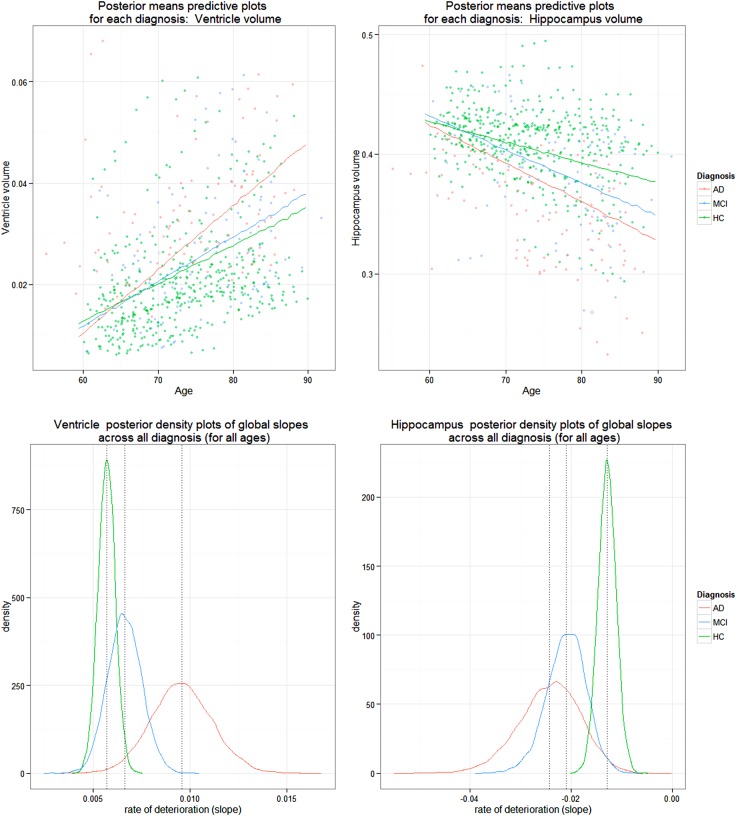
Posterior densities of population mean estimates of linear deterioration rate for diagnosis (top plot): HC, MCI and AD, for ventricle (left) and hippocampus volume (right) models. Dotted lines on bottom plots denote the means for each density, whose values are shown in table 1. AD, Alzheimer's disease; HC, healthy control; MCI, mild cognitive impaired.

The atrophy patterns for the ventricle and hippocampus regions described in the AIBL longitudinal study of ageing section are reflected in the results of the BLME models. A decrease in hippocampus volume and an increase in ventricle volume is depicted by the posterior densities for the rates of deterioration for the two responses as shown in figure 2. As expected, this biological pattern across the three levels of diagnosis is reflected in figure 2 as well as in tables 1 and 2. The ventricle population estimates of deterioration show an increase in volume as the diagnosis progressively worsens and the hippocampus population estimates of deterioration reflect a decreasing negative slope from HC, to MCI and AD. The overlapping densities are expected as individuals generally progress gradually in order of deterioration from HC to MCI to AD. Despite this overlap, there are distinct differences between the average rate of volumetric deterioration between the three diagnoses, as seen in table 1.

**Table 1:** Posterior means for rates of deterioration across three diagnosis levels for ventricle and hippocampus volume (top), and group differences among the three diagnosis levels (bottom), credible intervals for estimates in parentheses

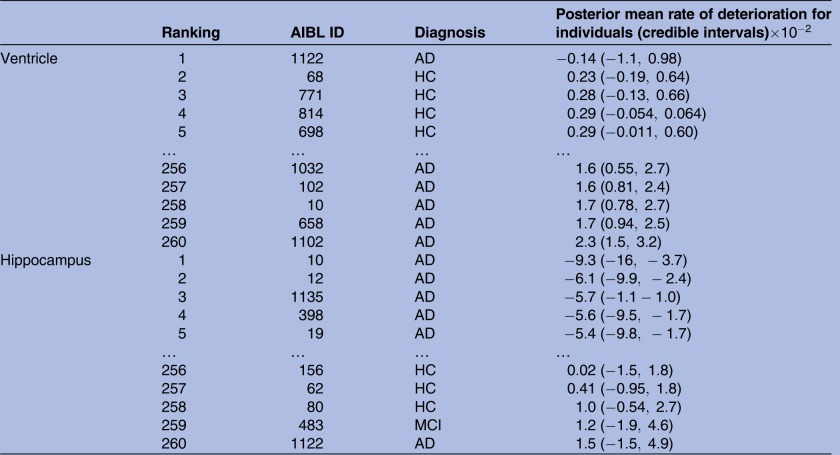

AD, Alzheimer's disease; HC, healthy control; ICV, intracranial volume; MCI, mild cognitive impaired.

**Table 2:** Posterior probabilities showing comparisons between HC, MCI and AD for ventricle and hippocampus volume

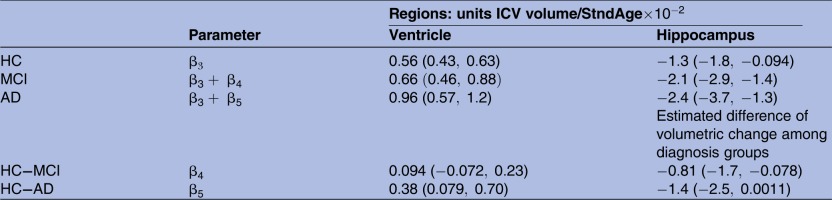

These results provide strong evidence regarding the order of diagnosis levels, derived from the case study.

AD, Alzheimer's disease; HC, healthy control; MCI, mild cognitive impaired.

**Figure 3:**
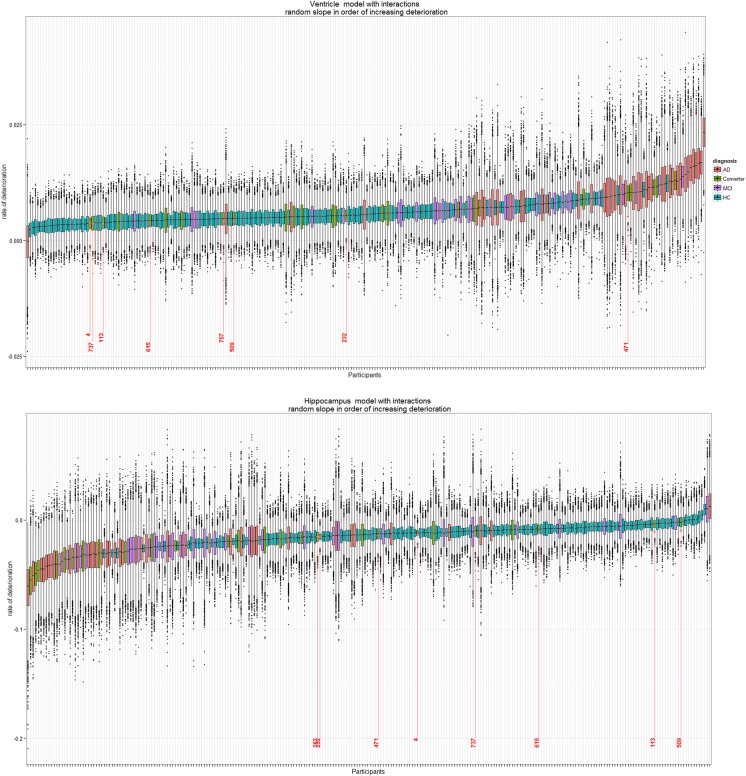
Box plots of posterior distribution of random-effect values for participants in the AIBL study (N=260) for full data (four time points). Ventricle (top) and hippocampus (bottom) rates of deterioration for each participant in the study. Since there are 157 HC, 34 MCI, 42 AD and 27 converters in this study, there is a higher uncertainty on the rate of deterioration of converters, MCI and AD participants (hence longer box plots) as compared with the HC (narrower box plots). Eight individuals who converted from HC to MCI throughout the study are highlighted in red with corresponding ID numbers. AD, Alzheimer's disease; AIBL, Australian Imaging Biomarker and Lifestyle Study of Ageing; HC, healthy control; MCI, mild cognitive impaired.

Tables 1 and 2 present estimated rates of change as well as the probabilities of diagnosis ordering for the hippocampus and ventricles. Furthermore, the difference among HC and degeneration levels MCI and AD shows the additional annual standardised age rate of change from baseline. The increasing range of the credible intervals for each group as deterioration progresses from HC to MCI to AD illustrates the stratified structure of different sample sizes over groups in our data. The box plots in figure 3 also demonstrate the general variability due to various diagnosis sample numbers.

Our BLME models also allow for probability statements to be made, based on whether any of the slopes are greater or smaller than a biologically meaningful constant or threshold. Table 2 shows the posterior probabilities of deterioration ordering for the three diagnosis categories for ventricle and hippocampus volume, as computed in equation (4). The large probabilities support the sequential pattern of deterioration for both regions.

Group comparisons are generally done via hypothesis tests in a classical LME and do not allow for probability statements of group ordering or provide visualisation on the distribution of the three groups which quantify the variability in the varying group sizes. The results presented in table 1 support our hypothesis test results (full analysis in online supplementary material), which show that MCI and AD slopes were significantly different from baseline for the hippocampus model, whereas only AD slope was significant for the ventricle model, while both classical and Bayesian results can be compared in tables 1 and 2 and the bottom of figure 2 can only be produced under the Bayesian framework.

### R: How to identify individuals with high levels of neurodegeneration?

The rates of deterioration (as measured by the rate of change with respect to age) for the ventricle and hippocampus are in reverse order; large positive ventricle slopes denote high atrophy, whereas low negative slopes denote large hippocampus atrophy. Table 3 shows a snippet of the participants ranked in order of their estimated median posterior deterioration rate. The data available in this study are highly unbalanced; nonetheless, all individuals are ranked despite 19 patients being observed at a single time point only. This is due to the ‘borrowing strength’ aspect of mixed-effects models, in that information across all time points contributes to the estimation of the population trends.

**Table 3:** Ranking of individuals from largest to smallest in order of posterior expected rate of deterioration (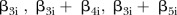) slope for all 260 participants, with 95% credible intervals in parentheses

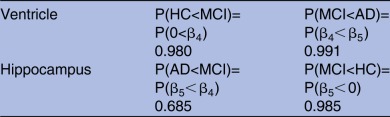

A snippet of the table shows the first and last five individuals, for ventricle and hippocampus volumes. Diagnosis levels: HC, MCI, AD and converter (either from HC to MCI, HC to AD or MCI to AD), to identify the 27 individuals who changed diagnosis throughout the study, as seen in figure 3.

AD, Alzheimer's disease; AIBL, Australian Imaging Biomarker and Lifestyle Study of Ageing; HC, healthy control; MCI, mild cognitive impaired.

Figure 3 shows clusterings based on HC, MCI and AD participants, denoted by the blue, purple and red box plots, respectively. This reflects the general order of diagnosis rates of deterioration for the ventricle and hippocampus volumes as shown in figure 2. However, there are a few individuals who do not follow this pattern, namely those in the small clustered group with the positive estimated levels of atrophy in the hippocampus model and participant ID 1122 in the ventricle model. Participant IDs 1122 and 483 are 2 out of the 19 individuals who only had baseline measurements, so the rate of deterioration was not observed, but it was still estimated. The same analysis was conducted with a classical LME model and figure 3 and table 3 were replicated (refer to the online supplementary material). We found strong similarities with the ranking of the eight converters of interest on hippocampus and ventricle models.

There were 27 individuals who progressed from baseline to a worse diagnosis. Eight individuals of interest are those who progressed from HC to MCI and who had at least three repeated observations recorded. Their estimated deterioration rankings are shown in figure 3. The majority of the eight converters in the hippocampus model are scattered along the lower half of the ranking of deterioration. This suggests that their linear rates of hippocampus neurodegeneration are less than those of the patients with AD. However, patient IDs 757, 232 and 471 were ranked approximately midway in this analysis, suggesting that they are approaching hippocampus rates of deterioration similar to AD, and out of the eight converters, they are the ones most at risk.

Likewise, for the ventricle model at the top of figure 3, patient ID 471 shows a ventricle rate of deterioration strongly similar to the AD cohort. Further investigation of patient ID 471, such as family mental history of other forms of dementia, stroke or other mental illness, current cognitive status and other health-related factors, may provide further insight as to why this individual has an unusually high rate of ventricle deterioration in comparison with the rest of the HC to MCI converters.

**Figure 4:**
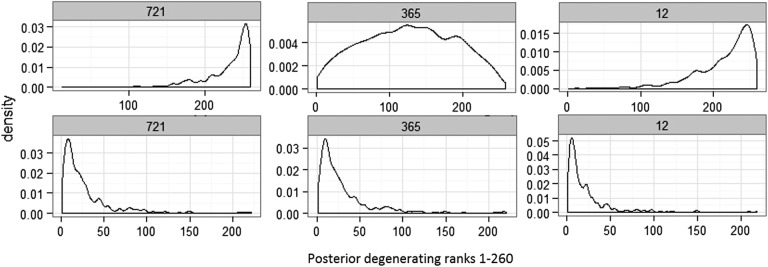
Posterior distribution of ranks for MCI to AD converters ID 721, 365 and 12, for ventricle (top) and hippocampus (bottom) ICV volume models. These density rankings were derived with observations from time points 1–3. AD, Alzheimer's disease; ICV, intracranial volume; MCI, mild cognitive impaired.

Figure 4 shows the posterior distribution of ranks for participant IDs 721 and 12 who converted from MCI to AD at time point 4. Probabilities of these individuals ranked in the lowest 15th quantile for the ventricle volume are 0.75 and 0.46, respectively, for participant IDs 721 and 12; likewise, for the hippocampus region, these probabilities are 0.47 and 0.58. This same analysis can be performed on any quantile range for any participants of interest. These probabilities show that these participants are in the high neurodegeneration extreme. These same analyses on the full data (over four time points) result in probabilities of participant IDs 721 and 12 ranked in the top 15th quantile are 0.80 and 0.66 for the ventricle and 0.54 and 0.69 for the hippocampus regions. Refer to the online supplementary material for posterior ranks distribution plots for all 27 converters. Under the classical implementation of the model in equation (3), the distribution of ranks for participants cannot be derived. Once participant ranking is estimated, no probability statements can be made to further analyse individuals at the high or low ranking extremes and compare, for example, the high and low 15th quantile extremes. Refer to the online supplementary material for the classical model results.

### R: How do diagnosis trajectories vary over age?

The aim of these analyses is to show the relationship between a volume centile, combined with results from external sources, to predict region-specific diagnosis changes over time. As described in the How do diagnosis trajectories vary over age? section, we present here the probability of a new individual diagnosed as either HC, MCI or AD conditional on volume range and specified age between 60 and 85 years.

Volume ranges, 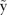, were the 75–100th, 50–75th, 25–50th and 15–25th centiles, as shown in figure 1 in the How do diagnosis trajectories vary over age? section. Equation (5) established relationships 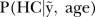, 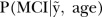 and 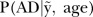, which consist of the output from the BLME model stated in equation (3) in conjunction with prevalence rates from Ward *et al*^52^ and Refshauge and Kalisch.^53^

**Figure 5:**
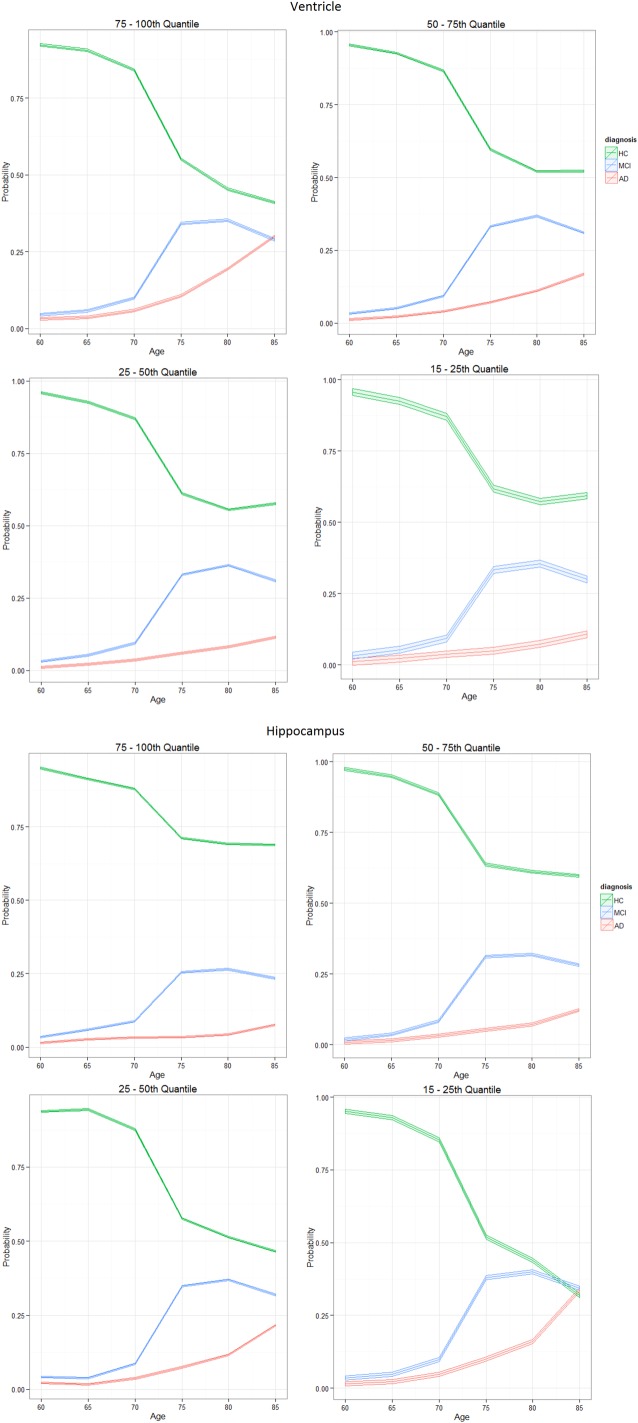
Probability curves show the posterior probability of HC, MCI or AD diagnosis for the ventricle (top) and hippocampus (bottom) models, while the 95% interval denotes the Monte Carlo error based on several simulations of the BLME models. Total volume is divided into four centile volume ranges, as shown in figure 1. Centiles: 75–100th, 50–75th, 25–50th and 15–25th. AD, Alzheimer's disease; BLME, Bayesian linear mixed-effects; HC, healthy control; MCI, mild cognitive impaired.

Uncertainty in the convergence trajectories of diagnosis levels is presented in terms of probabilities; hence, no credible intervals can be estimated. However, there is a Monte Carlo error associated with these estimates as they are derived from a finite sample from the posterior distribution. The ventricle and hippocampus models in equation (3) were estimated independently 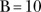 times; hence, every computation to derive the probability trajectories in this analysis was also estimated 10 times in order to compute the Monte Carlo SE estimates. Let the estimated quantity be denoted as 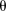 and 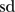 be the standard deviation; then a 95% interval for the Monte Carlo SE is estimated as 
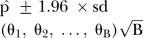
. As 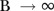, the Monte Carlo SE tends to 0, and while practically 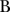 must be finite, our narrow CIs in figure 5 suggest that our simulation methods are adequate for our application.

The results in figure 5 show a large difference between HC in contrast with MCI and AD diagnosis for ages 60–75 across all ventricle volume quantiles. From age 75 onwards, those individuals in the top centile range (75–100th) show the quickest convergence of all the diagnosis levels, who by age 85 show an approximate equal probability (0.30 and 0.31) of being diagnosed as MCI or AD and only a slightly higher chance (0.39) of remaining HC. This contrasts those participants in the lower ventricle volume range (15–25th), whose difference in diagnosis is vastly different towards the later ages. By age 85, there is a mean estimated 0.60 probability of remaining HC, a 0.27 probability of being classified as MCI and an approximate 0.13 probability of AD diagnosis.

The hippocampus model results for this analysis are shown at the bottom of figure 5. Between the ages 60–70, there is very little difference across the diagnosis patterns, suggesting that individuals whose hippocampus volume lies above the 15th centile have an approximately equal risk of HC, MCI or AD diagnosis. From age 70 onwards, a noticeable difference in diagnosis trajectories is seen across all volume regions, 5 years earlier than the ventricle volume results. This is supported by a large body of literature,^5^ ^31^ ^57–60^ as the hippocampus is affected at an early stage of development of AD compared with other brain regions. Since a low hippocampus volume denotes high atrophy, individuals who fall in the lower range volumes, 15–25th centile, are most at risk of proceeding onto AD. Individuals in the lower hippocampus volume range, at age 85, have an approximate equal chance of HC, MCI or AD, as shown in figure 5.

Diagnosis trajectories over groups, male, female, APOE ε4 carriers and non-carriers, were also investigated for the hippocampus and ventricle regions using the model equation (3). We assumed the same prevalence rates within the population, for example, 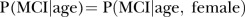; hence, the same broad prevalence rates from Ward *et al*^52^ and Refshauge and Kalisch 53 were used. Very little difference in the probable disease trajectory across all groups between ages 60 and 85 was observed (refer to the online supplementary material for plots). APOE 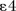 has been associated with an increased likelihood of developing AD.^61–63^ Gender differences regarding the prevalence of AD have also been studied.^64^ ^65^ Since the BLME models and inference derivation presented in this paper are the first of their kind, the objective of this analysis is to demonstrate probable diagnosis trajectories conditional on very broad, non-group-specific prevalence rates. Future models which account for APOE ε4, gender and other factors will use group-specific prevalence rates. However, to derive the same inference, this would require group-specific prevalence rates across ages 60–85, which are difficult to attain from the literature.

Our results support those presented in Holland *et al*,^15^ whereby diagnosis trajectories for neurodegenerated individuals (ie, those with a very low hippocampus and high ventricle volume) converge at the highest age group, in general over the age of 85. In particular, our results support those presented in Jack *et al*^30^ for a probabilistic trajectory of β amyloid negative and neurodegeneration-positive participants. To make our results comparable to those from Jack *et al*,^30^ HC participants whose hippocampus volume is less than the 50th centile are defined as neurodegeneration positive. While both methods present trajectories for neurodegeneration of participants over age, the BLME models presented here primarily estimate the rate of volumetric change for the ventricle and hippocampus regions. There are many other inferences that can be deduced from a combination of tapping into the vast wealth of AD research,^5^ ^66^ coupled with the present study analysis. The results presented here are some of the advantages of modelling neurodegeneration through mixed-effects models in the Bayesian framework.

## Discussion

In this research, we extended the level of insight commonly derived by LME models applied to longitudinal neuroimaging data into three key areas based on a BLME model on the ventricle and hippocampus ICV normalised volumes. We propose that a Bayesian approach for longitudinal neuroimaging modelling has merit for providing further understanding of brain atrophy over time. These views were demonstrated using an application of BLME models applied to a longitudinal AIBL study, which were compared with the classical alternative of LME models.

Comparisons of a volumetric rate of change of diagnosis-level trajectories were compared for HC, MCI and AD participants, with an estimated probability >0.65 on the order of disease pathology, while credible intervals for the parameters support results from the hypothesis test on a classical LME; under this framework, the probability of disease pathology order is not straightforward to compute. Ranking of converters with respect to the study cohort and diagnosis trajectories over age based on volumetric quantiles are the first BLME analysis of their kind applied to longitudinal neuroimaging data. This analysis identified HC to MCI converters most at risk of AD-like rate of deterioration and posterior rank distributions provided probabilities on individuals of interest in the worst 15th centile rank for both regions. The predictive capability of future converters can be derived from these BLME models, as individuals with high neurodegeneration estimates would rank at the extremes in comparison with the remainder of the cohort. The uncertainty of their rank values among a specified quantile is expressed in terms of probabilities, and individuals with a high probability of ranking at extreme levels of neurodegeneration may be indicative of their progressive pathway to further stages of dementia. While classical methods were also able to rank participants in order of estimated volumetric rate of change, they do not allow for further estimation of the highest ranked individuals and the uncertainty in their position. However, to rigorously validate this analysis, a richer data set with more repeated measures and converters over all categories (HC to MCI or AD and MCI to AD) observed at various ages is required. Furthermore, the diagnosis trajectories for each volume region identified critical points in time both ventricle and hippocampus degeneration from which participants are most likely to show greater deterioration rates. Alternatively, a similar analysis can also be used to compare diagnosis trajectories of different longitudinal neuroimaging population studies, such as the ADNI.

Additional analysis regarding group comparisons can be made. For example, similar probabilities for an estimated population mean in comparison to a biologically meaningful constant could also be inferred. An extension to our second analysis to allow population studies to focus on specific participants of interest and monitor their progression rate throughout follow-ups could assist health professionals in making informed choices with regard to patient care. Alternatively, HC to AD converters may also be further analysed and ranked with respect to the cohort, to provide further clues as to why these individuals deteriorated so quickly compared with their slower converter counterparts. It is worthwhile to note that these inference extensions would not have been possible had we not first attempted the research methods presented in this paper.

A sensitivity analysis with respect to the prior information used in our analysis was conducted on the ventricle and hippocampus models. This entailed rerunning the MCMC sampling technique for each model based on various specifications of the prior information. The subsequent posterior summaries did not vary considerably based on different prior information. Hence, we conjecture that the results are relatively robust to the priors specified in this work. Furthermore, two LOOCV methods were applied to assess the models’ predictive capability.

Despite every precaution taken to provide robust and reliable conclusions from the BLME models, several authors^7^ ^14^ have noted the limitations and disadvantages of Bayesian statistics applied to longitudinal neuroimaging analysis. In particular, drawbacks of Bayesian statistics in the neuroimaging context are discussed at length in Grunkemeier and Payne.^67^ These include subjective information that can be incorporated in the BLME model specification, in the way in which the prior is specified. Moreover, computational intensity is often far greater in the Bayesian framework than numerical methods employed in a frequentist analysis. In this paper, we incorporated vague priors which are semiconjugate, as we assumed no prior knowledge of the study analysed; the prior specification were a standard choice as suggested in Gelman and Hill.^23^ The additional computational time taken to run both models specified in the BLME in the context of the case study section was not excessive and was deemed to be worth the additional insight given. We suggest that more complex models and future extensions to the methods presented in this paper may result in an increase in computational time, and this will be a factor to consider in future BMLE models.

Extensions to the BLME models presented in this paper include the addition of more covariates to account for trends and variability sources present in gender, genetic factors and additional demographic characteristics which are a few of the key factors known to affect AD onset and disease progression. Furthermore, since the Bayesian framework is ideal for handling complex models such as generalised linear mixed models^41^ ^68^ and spatiotemporal interactions,^69^ ^70^ extensions of this nature will allow for modelling biomarker deterioration rates of multiple brain regions simultaneously over time.
